# Evaluation of two-stage designs of Phase 2 single-arm trials in glioblastoma: a systematic review

**DOI:** 10.1186/s12874-022-01810-7

**Published:** 2022-12-22

**Authors:** Wonsuk Yoo, Seongho Kim, Michael Garcia, Shwetal Mehta, Nader Sanai

**Affiliations:** 1 Ivy Brain Tumor Center, Department of Translational Neuroscience, Barrow Neurological Institute, Phoenix, AZ 85013 USA; 2 Karmanos Cancer Institute, Department of Oncology, School of Medicine, Wayne State University, Detroit, MI 48201 USA; 3 Department of Radiation Oncology, Barrow Neurological Institute, Phoenix, AZ 85013 USA

**Keywords:** Two-stage design of phase 2 single-arm trials in glioblastoma, Evaluation of Simon’s two-stage designs, Systematic review, Optiomal design, Minimax design, Adaptive design, Selection of historical control, Phase 0 trials, PRIMA

## Abstract

**Background:**

Due to economical and ethical reasons, the two-stage designs have been widely used for Phase 2 single-arm trials in oncology because the designs allow us to stop the trial early if the proposed treatment is likely to be ineffective. Nonetheless, none has examined the usage for published articles that had applied the two-stage designs in Phase 2 single-arm trials in brain tumor. A complete systematic review and discussions for overcoming design issues might be important to better understand why oncology trials have shown low success rates in early phase trials.

**Methods:**

We systematically reviewed published single-arm two-stage Phase 2 trials for patients with glioblastoma and high-grade gliomas (including newly diagnosed or recurrent). We also sought to understand how these two-stage trials have been implemented and discussed potential design issues which we hope will be helpful for investigators who work with Phase 2 clinical trials in rare and high-risk cancer studies including Neuro-Oncology. The systematic review was performed based on the Preferred Reporting Items for Systematic Reviews and Meta-Analysis (PRISMA)-statement. Searches were conducted using the electronic database of PubMed, Google Scholar and ClinicalTrials.gov for potentially eligible publications from inception by two independent researchers up to May 26, 2022. The followings were key words for the literature search as index terms or free-text words: “phase II trials”, “glioblastoma”, and “two-stage design”. We extracted disease type and setting, population, therapeutic drug, primary endpoint, input parameters and sample size results from two-stage designs, and historical control reference, and study termination status.

**Results:**

Among examined 29 trials, 12 trials (41%) appropriately provided key input parameters and sample size results from two-stage design implementation. Among appropriately implemented 12 trials, discouragingly only 3 trials (10%) explained the reference information of historical control rates. Most trials (90%) used Simon’s two-stage designs. Only three studies have been completed for both stages and two out of the three completed studies had shown the efficacy.

**Conclusions:**

Right implementation for two-stage design and sample size calculation, transparency of historical control and experimental rates, appropriate selection on primary endpoint, potential incorporation of adaptive designs, and utilization of Phase 0 paradigm might help overcoming the challenges on glioblastoma therapeutic trials in Phase 2 trials.

## Background

Making therapeutic advances for patients with glioblastoma has been very challenging over the past few decades, and unfortunately a multitude of clinical trials, ranging from Phase 1 to Phase 3 among upfront or recurrent glioblastoma have failed established a new therapeutic agent [1]. Recent research reported that the success rate in proceeding from Phase 2 to Phase 3 was the lowest among all three rates of Phase 1 to 2, Phase 2 to 3, and Phase 3 to approval (e.g., 29.7%, 20.3%, and 35.5% for oncology and 35.2%, 27.4%, 59% for overall) [2]. Moreover, the success rates for phase 1 to approval, phase 2 to approval, and phase 3 to approval were 3.4%, 6.7% and 35.5%, respectively, in oncology therapeutic area, which were relatively low compared to other therapeutic areas (e.g., 25.5%, 32.3%, and 62.2% of cardiovascular disease therapeutic area and 25.2%, 35.1%, and 75.3% of infectious disease therapeutic area) [3]. Wouters and colleagues reported that U. S. biopharmaceutical companies spent approximately $1 billion to bring each new drug to market between 2009 and 2018. The therapeutic areas in oncology and immune-modulatory drugs were the most expensive, with a median of $2.8 billion and a mean of $4.5 billion [3]. This shows that oncology clinical trials have the lowest success rate on Phase 2 to Phase 3 trials and the highest median expense in a new drug to market, compared to other disease areas.

Single-arm studies have been traditionally used in Phase II oncology clinical trials. Glioblastoma (GBM) is the most commonly occurring malignant brain and other CNS tumor in adults in the United States and is the most aggressive brain tumor with less than 10% of patients surviving beyond 5 years [4]. The rapid trials and ethical reasons generally lead the single-arm trials to be performed with interim analyses for possible early termination of the trials. Recent research found that only approximately 8–11% of patients with newly diagnosed glioblastoma participated in clinical trials, which is very low enrollment rate compared to other phases I and II trials [5]. Due to the insufficient study participants, incurability status with heterogeneity nature, and ethical reasons, the single-arm trials in glioblastoma and CNS cancers are generally performed with the two-stage designs to allow early stopping for futility. Simon’s two-stage designs have been widely used for single-arm trials in glioblastoma since Simon proposed his landmark paper in 1989 [6], and has been extended with various methodological development like the basket trials [7, 8] and Bayesian approach [9–11]. Particularly, a recent research of the phase 2 basket trials has extended the two-stage design for multiple heterogeneous indications, which is an important tool to identify the effective drug through a generalized framework of an optimal basket design. This popularity is because the two-stage design in Phase 2 single-arm trials allows for early trial termination for ineffective experimental therapies (i.e., futility). The two-stage design tests efficacy using the number of responses at the end of first stage of the trial and only if an efficacy threshold is met can the trial proceed to the second stage. If the therapy shows sufficient responses with the first stage data, the study will be continued for additional second stage with more patients to finalize the interim tests on the hypothesis that the therapy has sufficient biological activity to be able to advance for larger phase 3 randomized trials [6, 12]. In this manner, the two-stage designs allow stopping of a futile trial early. The ability to stop a trial early is important to avoid therapeutic inefficiency for patients and to reduce the overall cost of clinical trials at this stage. Therefore, the two-stage design in phase 2 trials provide a proof of concept that an experimental treatment is effective with small-sample efficacy evaluation before moving toward to bigger and confirmatory large-sample phase 3. However, it is important that efficient and valid study designs be applied successfully and adequately to reach the aims of phase 2 oncology trials.

Even though Simon’s two-stage designs have been popularly in oncology Phase 2 trials during last two decades, none, to our knowledge, has examined the usage for published articles that had applied the two-stage designs in Phase 2 single-arm trials in brain tumors. Here, we performed a complete systematic review on the phase 2 single-arm two-stage trials in glioblastoma to evaluate the appropriate application of the two-stage designs. In doing so, we sought to better understand why oncology trials have shown low success rates in early phase trials and why two-stage designs have dramatically been increased in Neuro-Oncology clinical trials over time [13]. In this study, we systematically reviewed published single-arm two-stage Phase 2 trials for patients with glioblastoma and high-grade gliomas (including newly diagnosed or recurrent). We also sought to understand how these two-stage trials have been implemented, and discussion of potential design issues which we hope will be helpful for investigators work with Phase 2 clinical trials in rare and high-risk cancer studies including Neuro-Oncology.

## Methods

### Search strategy

**Fig. 1:**
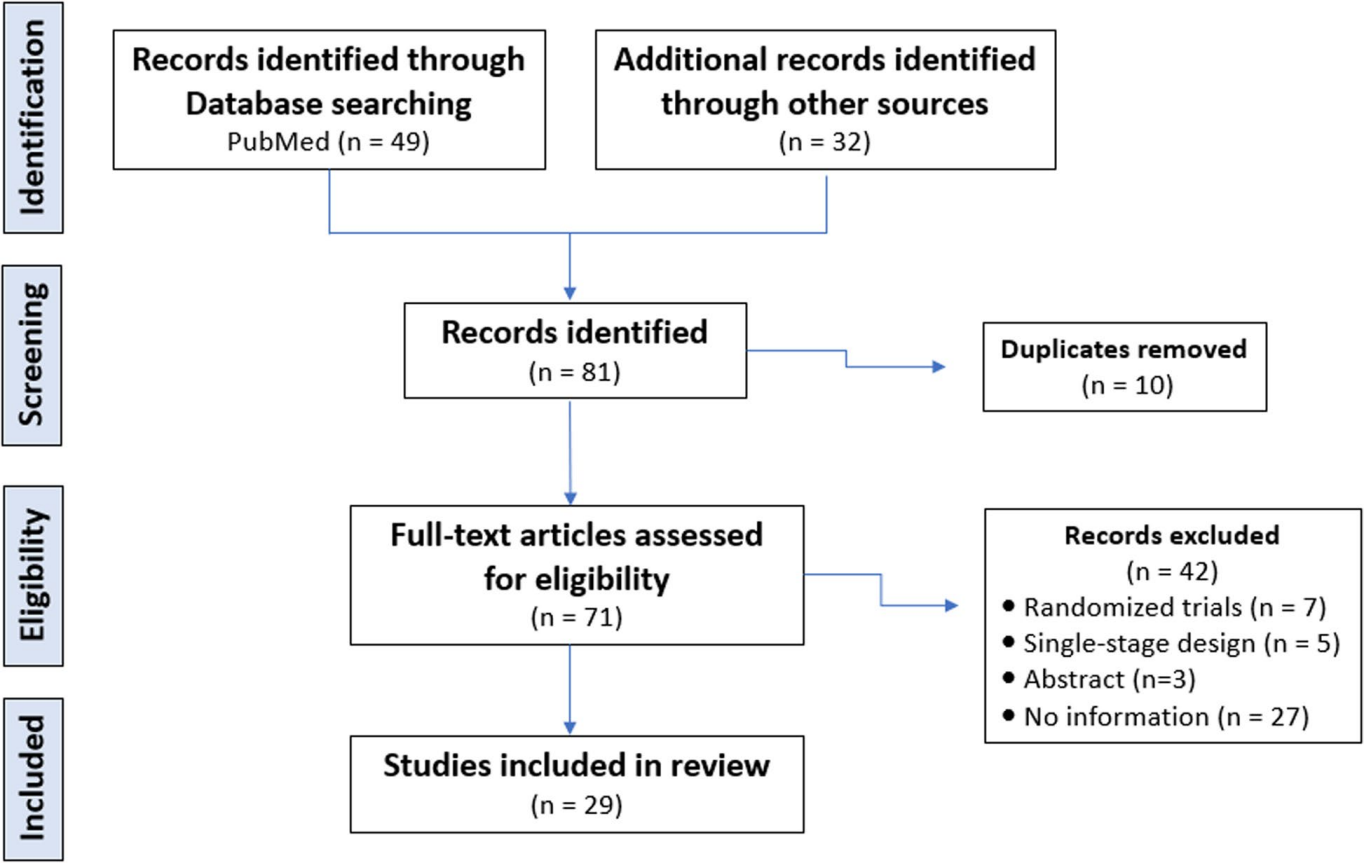
Flow diagram (PRIMA) of the literature search and study selection process

The systematic review followed the guidelines of the Preferred Reporting Items for Systematic Reviews and Meta-Analysis (PRISMA)-statement (http://www.prisma-statement.org) [14]. Searches were conducted using the electronic database of PubMed, Google Scholar and ClinicalTrials.gov for potentially eligible publications from inception by two independent researchers up to May 26, 2022. The followings were key words for the literature search as index terms or free-text words: “phase II trials”, “glioblastoma”, and “two-stage design”. The synonyms and closely related words include “phase 2″ for phase II trials, “GBM or high-grade glioma” for glioblastoma, and “2-stage, Simon, Fleming or Gehan” for two-stage design”. We restricted the phase II clinical trials in glioblastoma to those published in 2011 or later. There was no language restriction, but only complete papers published in peer-reviewed journals were considered. We identified a total of 81 articles based on online search using “Two-stage Phase II trials in glioblastoma”. Due to duplicates (*n* = 10), 71 articles were eligible to assess. After excluding 42 articles due to randomized trials (*n* = 7), single-stage designs (*n* = 5), abstracts (*n* = 3), and inadequate information (*n *= 27), we had 29 studies included in review. Figure 1 shows flow diagram (PRIMA) of the literature search and study selection process.

### Data extraction

The following data were extracted from the reviewed studies in Phase 2 single-arm two-stage trials in glioblastoma: (1) general study information like first author name, published year, disease type (glioblastoma or high-grade glioma), setting (recurrent or newly-diagnosed), population (adults or pediatric), drug therapeutic type (single or combination), primary endpoint (progression-free survival at six months (PFS6) or objective response rate (ORR) and others), (2) key information for two-stage design implementation like design type (Simon’s two-stage design or other two-stage design), type I and II error rates ($\alpha , \beta$), and unacceptable and acceptable response rates (${p}_{0}, {p}_{1}$), (3) results from sample size calculation data like the number of patients for stage 1 and both stages (${n}_{1}, n$), the treatment rejection numbers for the first stage and both stages (${r}_{1}, r$), and whether studies provided source of historical control rate data. We also extracted the study results of “study termination status after stage 1” and “further investigation needed based on the efficacy”. Simon’s two-stage designs include optimal and minimax design [6], while other two-stage designs include Fleming, Gehan, admissible two-stage design [15–17]. Table 1 shows the summary of the included studies for Phase 2 single-arm two-stage clinical trials in glioblastoma.

**Table 1:** Summary of the included studies for Phase II two-stage clinical trials in glioblastoma

First Author (Year)	General study design					Key information for Two-stage design implementation	
	Tumor type	Setting	Agent	Patient	PE	Design type	Type I Error
Chamberlain et al. (2011) [18]	GBM	Recurrent	BEN	Adult	PFS6	Simon (Minimax)	5%
Ananda et al. (2011)	GBM	Recurrent	TMZ + PLD	Adult	PFS6	Simon (Optimal)	10%
Neyns et al. (2011) [19]	Hight-grade glioma	Recurrent	SNT	Adult	Other	Simon (Minimax)	10%
Warren et al. (2012) [20]	Hight-grade glioma	Recurrent	TMZ + O6B	Pediatric	ORR	Gehan	NI
Geoerger et al. (2012) [21]	Hight-grade glioma	Recurrent	TMS	Pediatric	ORR	Simon (Optimal)	10%
Pan et al. (2012) [22]	GBM	Recurrent	SNT	Adult	PFS6	Simon (Minimax)	10%
Santoni et al. (2012) [23]	GBM	Recurrent	TMZ	Adult	PFS6	Simon’s Two-stage	10%
Hu et al. (2013) [24]	GBM	Recurrent	GMT	Adult	PFS6	Simon (Optimal)	5%
Lassen et al. (2013) [25]	GBM	Recurrent	BEV + TEM	Adult	Other	Simon’s two-stage	NI
Hargrave et al. (2013) [26]	Hight-grade glioma	Newly diagnosed	TMZ + IRI	Pediatric	ORR	Simon (Optimal)	5%
Tawbi et al. (2013) [27]	Brain metastasis	Newly diagnosed	TMZ + DAC	Adult	ORR	Admissible	10%
Muhic et al. (2013) [28]	GBM	Recurrent	NIN	Adult	ORR	Two-stage	5%
Burzynski et al. (2014) [29]	Hight-grade glioma	Recurrent	A10 + AS2-1	Pediatric	ORR	Two-stage	NI
Norden et al. (2015) [30]	Hight-grade glioma	Recurrent	NIN	Adult	PFS6	Simon (Optimal)	(A) 7.5% (B) 7.5%
Taylor et al. (2015) [31]	GBM	Recurrent	BOS	Adult	PFS6	Simon (Optimal)	5%
Lassman et al. (2015) [32]	GBM	Recurrent	DAS	Adult	PFS6 ORR	Simon’s two-stage	NI
Kalpathy-Cramer et al. (2017) [33]	GBM	Recurrent	TIV	Adult	PFS6	Simon’s Two-stage	NI
Arrillaga-Romany (2017) [34]	GBM	Recurrent	IMI	Adult	PFS6	Simon’s Two-stage	5%
Pellegatta et al. (2018) [35]	GBM	Newly diagnosed	TMZ + DEN	Adult	PFS12	Simon’s Two-stage	NI
Lee et al. (2019) [36]	GBM	Recurrent	BEV + PON	Adult	PFS3	Simon (Optimal)	10%
Silvani et al. (2019) [37]	GBM	Recurrent	ORT	Adult	PFS6	Simon (Optimal)	10%
Du et al. (2019) [38]	GBM	Newly diagnosed	TMZ + NIN	Adult	PFS6/OS12	Simon (Optimal)	5%
Sharma et al. (2019) [39]	GBM	Recurrent	DOV	Adult	PFS6	Simon’s Two-stage	10%
Kaley et al. (2019) [40]	GBM	Recurrent	PRF	Adult	PFS6	Simon (Optimal)	10%
Le Rhun et al. (2019) [41]	GBM	Newly diagnosed	THR	Adult	Other	Fleming	5%
Brenner et al. (2021) [42]	GBM	Recurrent	EVO + BEV	Adult	PFS4	Simon (Optimal)	5%
Altwairgi et al. (2021) [43]	GBM	Newly diagnosed	TMZ + ATO	Adult	PFS6	Two-stage	5%
Pasquualini et al. (2021) [44]	Hight-grade glioma	Recurrent	NIV + CYC	Pediatric	ORR	Simon (Minimax)	10%
Fangusaro et al. (2021) [45]	Hight-grade glioma	Recurrent	POM	Pediatric	ORR	Simon (Optimal)	5%

Key information for Two-stage design implementation			Results from sample size calculation				Study results	
Type II error	response rate (p_0_)	response rate (p_1_)	Total (N)	Stage 1 (N1)	Stage 1 Rejection No. (R1)	Total Rejection No (R)	Stopped after stage 1?	Further investigation needed
19%	15%	25%	31	16	1	NI	Stopped	Ineffective
10%	40–50%	50–70%	40	21	NI	NI	Completed	Ineffective
10%	5%	NI	32	18	0	3	Stopped	Ineffective
NI	5%	17%	25	16	1	NI	Stopped	Ineffective
10%	8%	30%	25	12	1	3	Stopped	Ineffective
20%	10%	25%	31	16	1	5	Stopped	Ineffective
10%	10%	25%	NI	NI	NI	NI	Stopped	Ineffective
20%	15%	30%	55	19	3	NI	Stopped	Ineffective
20%	NI	NI	32	13	NI	NI	Stopped	Ineffective
20%	5%	20%	29	10	0	3	Stopped	Ineffective
15%	7%	21%	34	14	0	4	Completed	Effective
24%	10%	25%	32	16	3	NI	Stopped	Ineffective
NI	NI	10%	40	20	0	NI	Stopped	Ineffective
(A) 20% (B) 20%	(A) 36% (B) 20%	(A) 55% 40%	NI	14	NI	NI	Stopped	Ineffective
20%	9%	30%	30	10	1	5	Stopped	Ineffective
5%	11%	25%	77	27	2	NI	Stopped	Ineffective
20%	NI	NI	18	5	0	NI	Stopped	Ineffective
16%	10%	30%	30	17	NI	NI	Stopped	Ineffective
NI	27%	42%	74	24	7	NI	Stopped	Ineffective
20%	15%	35%	27	15	4	9	Stopped	Ineffective
10%	20%	35%	58	33	6	NI	Stopped	Ineffective
10%	66%	87%	35	NI	NI	NI	Completed	Effective
20%	36%	55%	32	19	7	14	Stopped	Ineffective
10%	5%	20%	37	12	0	3	Stopped	Ineffective
5%	10%	35%	31	20	2	6	Stopped	Ineffective
20%	10.9%	28.9%	33	11	1	6	Completed	Effective
20%	55%	75%	32	15	NI	NI	Completed	Ineffective
10%	10%	30%	25	12	1	5	Stopped	Ineffective
10%	10%	40%	20	9	1	4	Completed	Ineffective

*GBM* Glioblastoma, *PE* Primary endpoint, p_0_ Unacceptable response rate, p_1_ Acceptable response rate, *Glioma*^a^: high-grade or malignant glioma, *NI* No information, *BEN* Bendamustine, *TMZ* Temozolomide, *PLD* Pegylated liposomal doxorubicin, *SNT* Sunitinib, *O6B* O6-benzylguanine, *BEV* Bevacizumab, *RLT* Rilotumumab, *TMS* Temsirolimus, *GMT* Gimatecan, *TEM* Temsirolimus, *IRI* Irinotecan, *DAC* Decitabine, *NIN* Nintedanib, *A10* + *AS2-1* Antineoplastons A10 and AS2-1, *SOR* Sorafenib, *TIV* Tivozanib, *BOS* Bosutinib, *DAS* Dasatinib, *IMI* Imipridone ONC201, *DEN* Dendritic, *PON* Ponatinib, *ORT* Ortatzxel, *NIM* Nimotuzimab, *DOV* Dovitinib, *PRF* Perifosine, *THR* Thrombopoietin receptor, *EVO* Evofosfamide, *ATO* Atorvastatin, *NIV* Nivolumab, *CYC* Cyclophosphamide, *POM* Pomalidomide

### Assessment of two-stage designs

Using four key input parameters of two types of error ($\alpha , \beta$) and two hypothesis rates of unacceptable maximum response rate of historical control (${p}_{0}$) and acceptable minimum response rate of study expectation (${p}_{1}$), we can implement the sample size calculation of the two-stage designs. As output results, the two-stage designs (Optimal, Minimax, and Admissible designs) produce following key outputs of the number of patients (${n}_{1}$ and ${n}_{2}$) for stage 1 and both stages and the rejection numbers (*r* and ${r}_{1}$) for both stage 1 and both stages respectively. Thus, the key input parameters ($\alpha , \beta , {p}_{0}, {p}_{1}$) and output results (${n}_{1}, n, {r}_{1}, r$) were investigated to assess the appropriate usage and report of the two-stage phase 2 trials. Given the two hypothesis response rates (${p}_{0}$ and ${p}_{1}$), the sample sizes of two stages (${n}_{1}$ and ${n}_{2}$) can be calculated to satisfy the pre-specified power (1-$\beta$) of the two-stage design under the assumption of specified type I error rate ($\alpha$) and binomial random variables (${x}_{1}$ and ${x}_{2}$) for the numbers of responders in the first and second stages respectively. Suppose a two-stage design with a type I error no larger than ${\alpha }^{*}$ and a power no smaller than (1- ${\beta }^{*}$) for given (${p}_{0}$, ${p}_{1}$). If the parameters of two-stage designs (${p}_{0}, {p}_{1}$) are given, there are infinitely many two-stage designs satisfying the (${\alpha }^{*}, {\beta }^{*})$ condition. Finally, we can calculate the expected sample sizes of ${n}_{1}$ or $n$ with a true response rate of the experimental therapy since the sample size of ${n}_{1}$ and $n$ are random variables [6, 12, 17]. Since the two-stage design of Phase 2 trials aims to make a conclusion of go or no-go to the next second stage, the sample size calculation of two-stage designs produces the number of patients in first stage (${n}_{1}$) and the response number which is eligible to move to the second stage (${r}_{1}$). If advanced to stage 2, the number of patients in both stages ($n$) and the response number ($r$) of the efficacy for the Phase 2 trial are provided.

## Results

### Overall evaluation

**Fig. 2:**
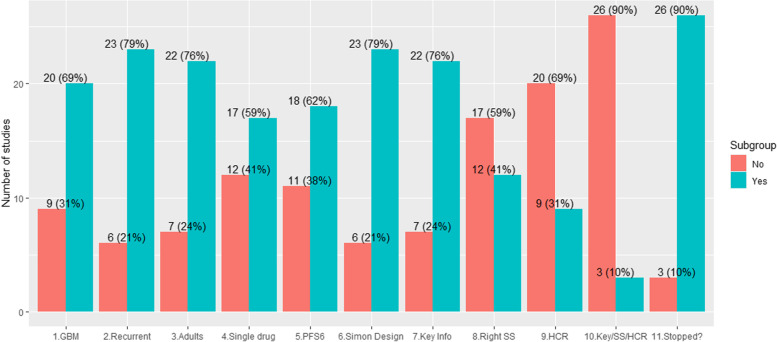
Results of design input parameters and sample size calculation output from reviews of Phase 2 single-arm two-stage designs in glioblastoma. GBM: glioblastoma, PFS6: progression-free survival at 6 months, Key info: key input parameters for two-stage design, Right SS: did two-stage sample size calculation be appropriately implanted? and HCR: did the reference of historical control rate be provided?, all key input and output information as well as reference of historical control rates provided (Yes, No)?, and (11) did the trial be stopped (Yes, No)?

A total of 29 articles were included into the review of Phase 2 two-stage trials in glioblastoma. Among 29 reviewed articles, majority study types were glioblastoma (*n* = 20, 69% over high-grade glioma, *n* = 9, 31%) with recurrent patients (*n* = 23, 79% over newly diagnosed patients, *n* = 6 and 21%) and adult patients (*n* = 22, 76% over pediatric population, *n* = 7, 24%). Table 1 is the summary of the included studies [18–46]. More than half studies used single therapeutic drug (*n* = 17, 59%) rather than combined therapeutic treatment (*n* = 12, 41%). 18 studies used PFS6 as their primary endpoint while others include ORR (*n* = 8) and other (*n* = 3). Except for three clinical trials that didn’t provide the methods used, almost all articles were Simon’s two-stage designs (*n* = 23, 90%). The other three trials used two-stage designs like Inadmissible design, Fleming and Gehan designs. Study design input information and output results from sample size calculation related to two-stage design implementation were examined. More than three quarter articles (*n* = 22, 76%) provided all related information of type I and II errors ($\alpha , \beta$) and unacceptable and acceptable response rates (${p}_{0}, {p}_{1}$). But interestingly almost 60% of studies (17/29, 59%) failed to provide at least one key output results of sample size calculation such as the number of samples of first stage and both stages (${n}_{1}, n$) and the treatment rejection numbers of the first stage and both stages (${r}_{1}, r$). Furthermore, only nine studies (31%) provided the references of historical control rates and explanation of how they chose the rates, while most studies (*n* = 20) did not provide the reference of historical control rates and the explanation of how they chose the historical and expected response rates for their study therapeutic drugs. Only three trials (11%) provided key input parameters, appropriately reported output results from sample size calculation of two-stage designs, and finally provided the reference and explanation of historical control rates. Among 29 trials, only three has been completed for both stages and two studies have shown the efficacy. Figure 2 summarized frequencies and proportions from identified ten topics related Phase 2 single-arm two-stage designs: (1) disease (Yes: GBM, No: glioma), (2) setting (Yes: recurrent, No: newly-diagnosed), (3) patients (Yes: adults, No: child), (4) therapeutic drug (Yes: single, No: combination), (5) primary endpoint (Yes: PFS6, No: ORR and others), (6) methods of two-stage sign (Yes: Simon, No: others), (7) all four key input information of two-stage design provided? (Yes, No), (8) all four output results of sample size appropriately reported? (Yes, No), (9) reference of historical control data provided? (Yes, No), (10) all key input and output information as well as reference of historical control rates provided (Yes, No)?, and (11) did the trial be stopped (Yes, No)?

### Summary for general study design

Most frequently used population was adult patients with recurrent glioblastoma. Disease population was categorized into three diseases of glioblastoma (*n* = 20), high-grade glioma (*n* = 8), and brain metastasis from glioblastoma (*n* = 1), two settings of recurrent status (*n* = 23) and newly diagnosed status (*n* = 6), two patient types of adults (*n* = 23) and child or pediatric (*n* = 6), and two therapeutic drug types of single (*n* = 17) and combination (*n* = 12). Temozolomide (TMZ) was mostly used for combination therapeutic drugs (*n* = 7 with pegylated liposomal doxorubicin (PLD), O6-benzylguanine (O6B), irinotecan (IRI), decitabine (DAC), Dendritic (DEN), Nintedanib (NIN), and Atorvastatin (ATO)) while Bevacizumab (BEV) was second mostly used for combination drugs (*n* = 3 with temsirolimus (TEM), Ponatinib (PON), and Evofosfamide (EVO)). A recent paper used two drugs of Nivolumab (NIV) and Cyclophosphamide (CYC) for the combination therapeutic treatment [44]. A total of 17 drugs were used as single therapeutic treatment with Sunitinib (SNT) and Nintedanib (NIN) from two studies each, and Temozolomide (TMZ), Bendamustine (BEN), Temsirolimus (TMS), Gimatecan (GMT), Bosutinib (BOS), Dasatinib (DAS), Tivozanib (TIV), Imipridone (IMI), Ortatzxel (ORT), Dovitinib (DOV), Perifosine (PRF), Thrombopoietin receptor (THR), and Pomalidomide (POM) from single study each. The most widely used endpoints were PFS6 (*n* = 18) and ORR (*n* = 8) in Phase 2 single-arm trials.

### Key input information for two-stage design implementation

Among 29 Phase 2 single-arm trials, 23 trials (79%) used Simon’s two-stage designs, three trials used other two-stage designs (Gehan, Fleming and admissible design each), and 3 trials just mentioned two-stage design without specific design information. Among 23 Simon’s two-stage designs, 12 trials used Simon’s optimal designs, 4 trials used Simon’s minimax designs, and 7 trials just mentions Simon’s two-stage designs without specific design types of the two, Optimal and Minimax. It’s interesting to see that most trials without mentioning specific design types (like Simon’s optimal or minimax, Gehan, Fleming, or admissible designs) failed to provide one or more than one key information for the implementation results of two-stage design sample size calculation. The two types of errors ($\alpha , \beta$) and unacceptable and acceptable response rates (${p}_{0}, {p}_{1}$) are key input information for successful sample size calculation of two-stage design. Most trials (*n *= 22, 76%) successfully provided all key information while 7 trials (24%) failed to provide at least one key information (Six trials failed to provide two types of error rates, four trials failed to provide two response rates, and three trials failed to provide two or more than two key results from the sample size calculation).

### Key output results from two-stage design sample size calculation

Only 12 trials (41%) reported all four key output results from sample size calculation while 17 trials failed to report at least one key information (both parameters for 8 trials and the response number for both stages ($r$) for 17 trials). Most studies (*n* = 27, 93%) provided the number of patients in stage 1 and both stages, so many trials (*n* = 17) failed to report one or more from both response numbers of stage 1 and both stages which are key information to determine the study continuation toward the second stage (${r}_{1}$) at the end of first stage and hypothesis testing of efficacy ($r$) at the end of second stage. Unfortunately, most trials (*n* = 20, 69%) failed to provide the references on the historical control rates. Furthermore, all trials except one trial did not explain how they chose the acceptable response rate. Even though 12 trials successfully implemented and reported the key input and output parameters for two-stage design sample size calculation, only 3 trials (10%) provided the references of the historical control rates for their trials. Regardless that more than 75% trials mentioned all key input parameters, many studies (17/29, 59%) failed to provide at least one key output of sample size calculation results of the number of samples of both stages (${n}_{1}, n$) and the treatment rejection numbers of the first stage and both stages (${r}_{1}, r$). In addition, the several trials provided wrong results from sample size calculation even if they reported all related key information for two-stage design implementation (not shown in table). Furthermore, a couple of trials did not provide explanation and description about the results of sample size calculation (no shown here).

## Discussion

In this systematic review, we have examined 29 studies from Phase 2 single-arm two-stage trials in glioblastoma to assess the appropriateness and transparency of the study design and sample size calculation of Phase 2 single-arm two-stage trials in glioblastoma. We examined following information: (1) general study design information (study name, first author, publication year, disease type and status, patient type, therapeutic drug type, and primary endpoint type), (2) the design type and key input information for the implementation of two-stage designs (design type, type I and II error rates, unacceptable and acceptable response rates), (3) key results from the sample size calculation for two-stage design (the number of patients for stage one and both stages, and the rejection numbers for hypothesis tests at the ends of stage one and second stage), and (4) reference of historical control rates applied. Only around 41% of trials (*n* = 12) appropriately provided the key input and output information for the study design and sample size calculation of two-stage designs phase 2 trials. Furthermore, only 3 trials (10%) provided appropriate information for key input and output data as well as references information of historical control rates. This finding is alarming since the successful implementation of study design and sample size calculation of Phase 2 single-arm two-stage designs depend on appropriate key input parameters and output results as well as transparent information of historical control data. Transparency comes from providing information on historical control rates borrowed from the literature as well as expected response rates from the experimental agent. Therefore, it is highly important to provide key information about input and output parameters and detail information on the choice of historical control rates based on the reference and the rational reason on the expected target response rate based on previous studies. Several important topics related to the design issues will be followed to handle the design issues by increasing the precision of efficacy from targeted therapeutic trials of Phase 2 two-stage designs in brain tumor.

### Determination of historical control rates

**Fig. 3:**
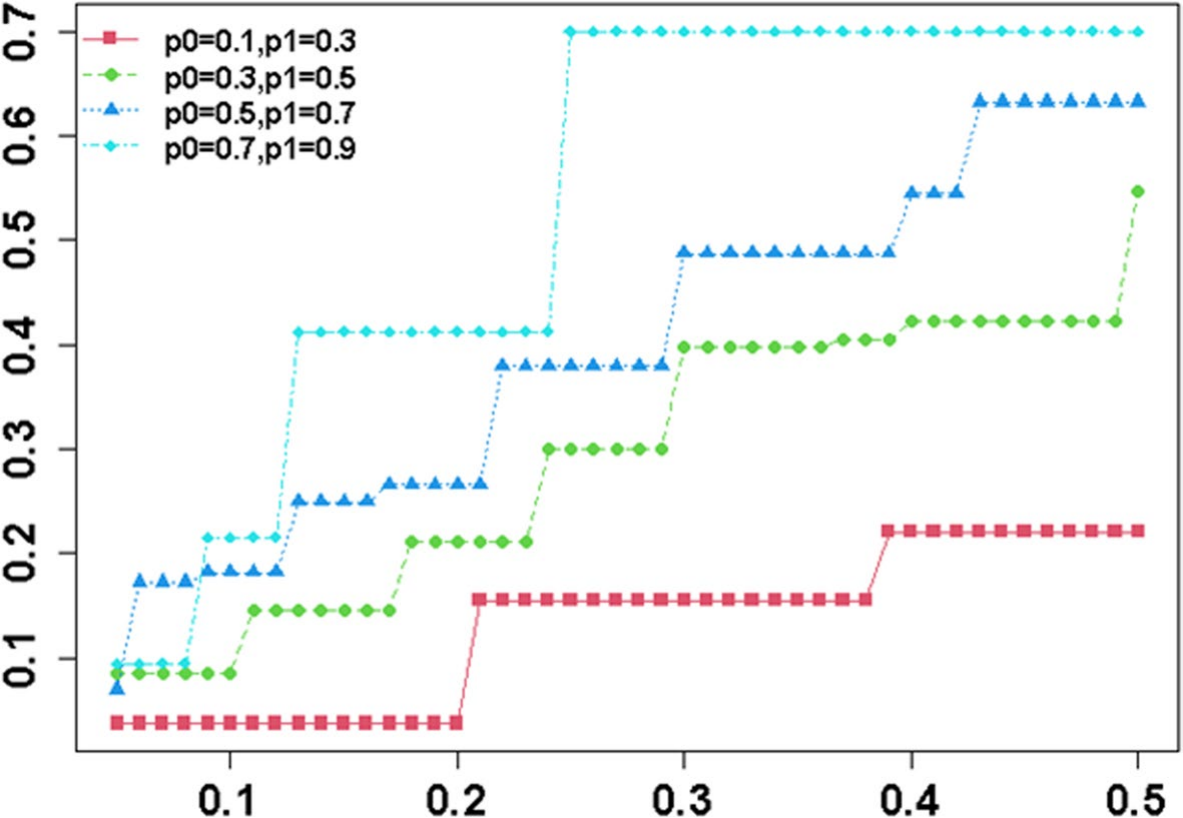
Loss of power according to the underestimation rate of a null hypothesis (p0) when a single-arm single-stage design is used for a binary endpoint Under 80% of power and 5% of one-sided Type 1 error rate. The x-axis is underestimation rate of a null hypothesis and y-axis is loss of power from the power of 80%

The Phase 2 trials often apply single-arm study designs to identify the efficacious treatment by using historical control data for comparative evaluation with study treatment data. The sample size calculation for the two-stage designs is determined based on the historical control rate and difference between the two rates (${p}_{0}, {p}_{1}$). Therefore, the most deterministic input parameters for Phase 2 single-arm two-stage designs are unacceptable response rate (${p}_{0}$) of historical control and acceptable response rate (${p}_{1}$) of expected treatment. The unacceptable historical control rate should be a maximum rate while the acceptable expected treatment rate should be a minimum rate in order not to be overly optimistic for the Phase 2 clinical trials. Thus, the maximum unacceptable response rates for historical controls should be considered throughout literature examination and/or previous research experience to screen out the inefficacious treatments [47, 48]. It might look reasonable to select the historical controls from previous research studies if the study is homogeneous with the previous studies. However, we should acknowledge there exist heterogeneities when we borrow the historical control information from other published studies due to different population conditions and quality of supportive care. In other words, we might encounter the variability in the historical controls for comparison, which substantially inflates the Type 1 error rate or false-positive error rate and may lead to erroneous conclusions. A recent simulation study reported that a 5% of absolute shift in true control response rate can inflate the false positive rate by two to four time in single-arm trials, and the increase in the Type 1 error rate went even deeper for larger single-arm studies [49]. To illustrate the effect of underestimated historical control on the study power, we carried out a simulation study as follows. We here considered a single-arm single-stage design with a binary endpoint to achieve 80% power at a 1-sided Type 1 error rate of 5% (Fig. 3). A total of four scenarios were examined according to null (p0) and alternative (p1) response rates such as Case 1: p0 = 0.1 and p1 = 0.3, Case 2: p0 = 0.3 and p1 = 0.5, Case 3: p0 = 0.5 and p1 = 0.7, and Case 4: p0 = 0.7 and p1 = 0.9. The underestimation rate of p0 was ranged from 0.1 (i.e., 10% reduction) to 0.5 (i.e., 50% reduction). For instance, in Case 3, the 10% and 50% reductions of p0 are 0.45 (= 0.5 × 0.9) and 0.25 (= 0.5 × 0.5). The outcomes of simulation are depicted in Fig. 1. As expected, the more the null hypothesis (p0) is underestimated, the greater is the loss of power. To avoid this temptation, which poses a potential risk to patients, investigators should practice transparency by citing historical data sources used in the study protocol. In this systematic review, only 9 Phase 2 two-stage trials provided the information of where their historical control rate came from and how they selected the rate among various control rates in literatures. Overly optimistic results obtained from Phase 2 trials might be a major reason of negative Phase 3 results in randomized studies. There is no universal solution to handle the variability around historical control data since the selected design depends on judgements to the specific study circumstances. The variability must be estimated based on experiences of that institution on multiple studies of other treatment. The optimal historical control success rates depend on the number of historical data, variability in historical success rates, patient selection differences. These factors are recommended to be considered carefully when planning a Phase 2 single-arm study.

### Type 1 and 2 error rates

Two types of errors ($\alpha , \beta$) are key information for all study designs. Phase 2 single-arm trial designs typically allow Type 1 and 2 error rates up to 20% [50]. The choice of type I and type II errors is highly important since researchers need to assure that potentially effective therapy is not abandoned and at the same time the insignificant studies with very small marginal effects should not be advanced to subsequent phase III studies. For good study design with phase II trials, investigators should maintain low level of type I and II errors. How low levels for both errors are low enough to be a good design? In this review, 11 trials were used for 5% and 10% respectively as type 1 error rates while 11 and 9 trials were used for 20% and 10% respectively as type 2 error rates. And 7 trials were used with (1) 5% and 20% (2) 10% and 10% as their type 1 and 2 errors respectively. Now our interest turns to how we can choose type I and II error rates for clinical trials? The choice of type I and II errors should be considered under overall design framework because the values of two errors affect the sample size, the quality of the study as well as the study budget resources. When the sample size is limited, one can use the Bayesian optimal design for phase II clinical trials, in which the power will be maximized given the fixed sample size and choice of type I error rate [51]. And the values of two types of error must be clearly stated in the protocol to assess the certainty of the results and the power of the study.

### The choice of two-stage designs: minimax or optimal?

Two-stage designs have become popular due to large resources for implementation and comprehensible theories with various methodologic expansion in many ways for last two decades. Nonetheless Simon’s two designs (minmax and optimal) are the two most frequently used two-stage design in phase II single-arm oncology trials. Which one should be chosen for our studies of the two? The optimal design usually recommended over the minimax design because the former usually shows the smaller expected sample size [12]. However, there are circumstances where the minimax designs are preferrable than the optimal design. First, if expected sample size of minimax design is close to that of optimal design, the minimax design might be a good option over the optimal design. Second, if the patient accrual rate is low, the minimax design is more attractive because it requires the smaller number of patients in total (n). Let us assume that the result of a two-stage design indicates 18 and 23 in total patients for the minimax and optimal respectively. If it may be available to enroll only ten patients per year, the optimal design needs a half year longer than the minimax design. Third, when expected sample sizes from both designs are close each other, the minimax design may be more appropriate than the optimal design because the minimax design produces the smaller total patients [6]. In summary, Simon’s 2-stage designs, under the same type 1 error rate and power, the minimax design has a smaller total sample size than the optimal design, while the optimal design has a smaller stage 1’s sample size than the minimax design. We can see that four and twelve studies utilized Simon’s minimax and optimal designs respectively, in Table 1. This implies that most brain tumor clinical trials are at high risk with great uncertainty in trial outcomes. Another option of two-stage designs is “admissible design” or “spatial design”, which came from an idea “Can we find a good alternative design between the minimax and optimal design. Jung et al. (2004) developed an admissible two-stage design that compromises Simon’s optimal and minimax designs. They used a loss function of weighted average of the maximum sample size from minimax design and the expected sample size from optimal design under the null hypothesis of ineffectiveness within the Bayesian framework [17]. Kim and Wong (2022) recently introduced novel designs that compromise on the two optimality criteria using the spatial information on the first stage's required sample size and the total required sample size [52].

### Primary endpoint: PFS is a good surrogate for OS?

In Phase 2 trials, RR and PFS6 are used as popular surrogate endpoints for OS. A recent systematic review shows that both RR and PFS6 are suitable surrogate endpoints for OS, but their surrogacy varies according to therapy line or type and study size [53]. Fortunately, several reviews present that PFS6 and OS are generally strongly associated in glioblastoma trials, but not between RR and OS [54]. Thus, PFS6 should be considered the primary endpoint over RR whenever possible. Alternatively, novel surrogates can be used instead of RR and PFS6. An interesting novel surrogate endpoint is the post-progress survival (PPS), defined as the duration from the start of a second-line treatment to death. Trippa and colleges [55] introduced a novel composite endpoint model by combining PFS6 and OS for glioblastoma trials. Their composite endpoint model provides efficiency while still maintaining the clinical relevance of OS. Wang et al. [56] proposed modified PFS (mPFS) for immune-oncology trials. mPFS does not include the events of disease progression but include the events of death within 3 months after randomization. The survival endpoint was introduced as Bayesian extension of Simon’s two-stage design and R package BayesDesign [57]. When there is uncertainty among endpoints as a surrogate for OS, several primary endpoints can be employed together. By doing so, the chance to capture the effectiveness of a treatment can be increased. Suppose both RR and PFS6 are considered primary endpoints. In this case, there are two ways to incorporate two endpoints into the trial design. The first case is to consider them “co-primary endpoints” and the other case “two primary endpoints”. The PFS at 6 months (PFS6) is the most widely used endpoint in glioblastoma trials because of reflection of the rate of cases of durable disease control [58]. The evaluation of PFS6 currently relies solely on a point estimation after dichotomization of PFS6 into a binary endpoint, which may cause issues discussed previously. A better way to avoid the potential issues is to use a hazard ratio (HR). Unlike a survival rate at a specific time point, a HR is not a point estimate and uses all the information in the entire survival curve. Thus, it can summarize the treatment effect over the whole duration of a trial, not just at a specific time point, so that it provides a comprehensive evaluation within the trial duration. Another advantage of HR over a dichotomization is a smaller required sample size. For instance, Silvani et al. [37] used Simon’s two-stage optimal design to evaluate the target PFS6 of 35% against the null PFS6 of 20% to achieve 90% power at a 1-sided 10% level, resulting in the required sample size of 58 patients. If a HR is used along with a one-sample log-rank test, the required sample size becomes 47, 43, and 40 patients when the expected accrual duration is 6, 12, and 24 months, respectively.

### Should we consider adaptive designs?

**Fig. 4:**
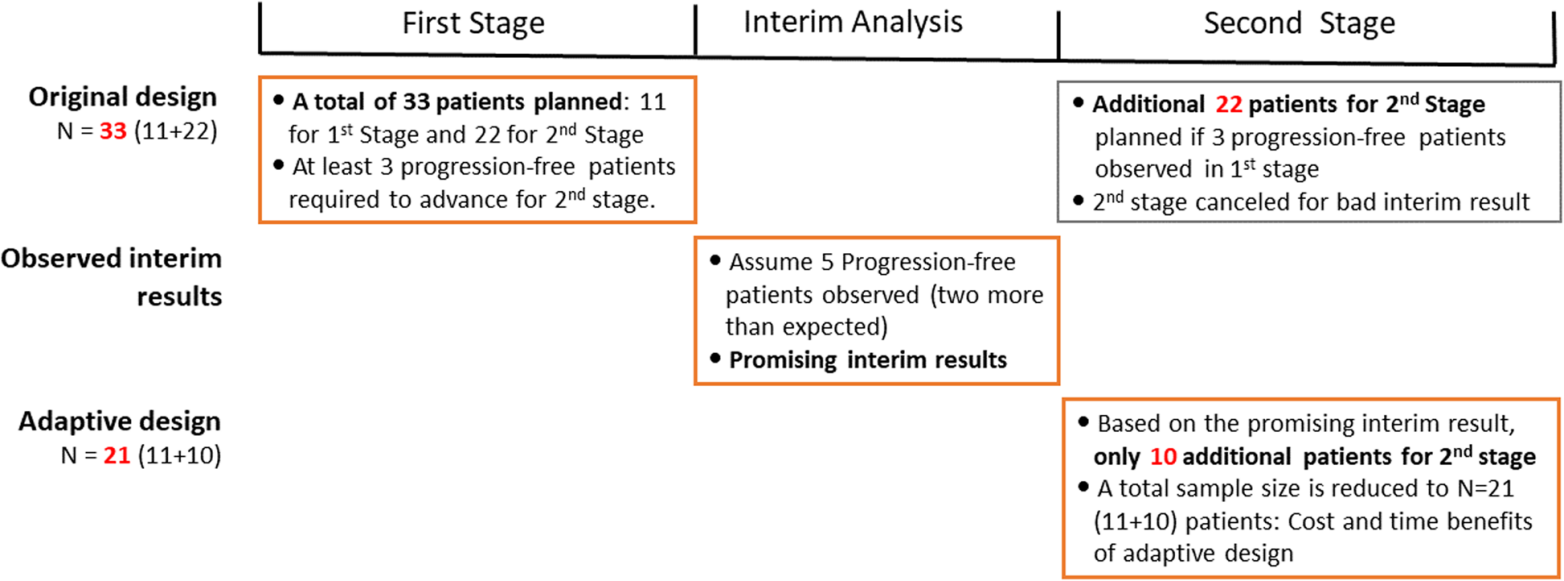
Example of Adaptive Design to handle the uncertainty for Phase 2 two-stage design. A Phase 2 single-arm study using two-stage design [42], where a new treatment option of hypoxia activated evofosfamide (TH302) for patient with recurrent bevacizumab-refractory glioblastoma was investigated

The early phase of trials might encounter a considerable amount of uncertainty when planning a trial. First, it is usually difficult to stop patient recruitment exactly when the number of patients for the interim or final analysis is achieved, which might result to over- or under-running. Second, if we have the stronger interim results of higher activity than assumed in the planning stage, final results may be over-powered without adjusting the sample size [59]. Such an unexpected situation cannot be appropriately handled with the current system of two-stage designs because current designs require to prespecify the design information like sample size for each stage and stopping rules in the study protocol. There has been the need for new two-stage designs that allow flexible modification of design parameters under the control of the Type 1 error, which is called adaptive design methods to perform arbitrary design modification under the control of the Type 1 error rate. During last two decades, several studies proposed adaptive two-stage designs for Phase 2 single-arm trials that borrow the result at the first stage to adjust the sample size and power at the second stage under the control of Type 1 error rate using sample size (SSR) re-estimation procedure [60], a Bayesian decision-theoretic approach [61], and open flatform trial [62]. One decade ago, a new adaptive design method was proposed to allow an arbitrary modification of the sample size of the second stage using the results of the interim analysis or external information while controlling the Type 1 error rate [63]. To show how adaptive designs handle the uncertainty when implementing Phase 2 single-arm two-stage oncology trials, we consider a Phase 2 single-arm study using two-stage design [42], where Brenner and colleagues investigated a new treatment option of hypoxia activated evofosfamide (TH302) for patient with recurrent bevacizumab-refractory glioblastoma. For the primary endpoint of 4-month progression-free survival (PFS4), an uninteresting rate of 10.9% obtained from historical controls and an anticipated rate of 28.9% were assumed. Simon’s optimal design for a one-sided significance level of 0.05 and a power of 0.80 includes 11 patients in the first stage. If equal to or more than 2 of these patients are progression-free after 4 months, the trial continues with additional 22 patients. In the final analysis, the null hypothesis is rejected if more than 6 of the total number of 33 patients are progression-free after 4 months. Let us now assume that 4 (36.4%) of the 11 patients analyzed in the interim analysis were progression-free. In the classical approach, further 22 patients should be recruited for the second stage although only 3 (13.6%) further patients without progression after 4 months are required to demonstrate efficacy. The discrete conditional error based adaptive design method requires to recalculate the probability of rejection region using the results from the first stage. Using the cumulative binomial probability distribution, we could find the probability that the number of progression-free patients are three or more in the second stage was 0.4357. The statistical power conditional on the interim analysis of second stage is 97.3% for the true rate of 28.9%, which is far beyond the originally pre-specified 80% of power. Assuming a true rate of 28.9%, additional 10 patients are sufficient to achieve 80% power. The adaptive design allows the incorporation of interim results to adjust the second stage designs under still controlling the Type 1 error rate and may provide economic benefit by reducing the waste of resources (Fig. 4).

## Limitations

This systematic review has some limitations. First, even though we performed a comprehensive search strategy, it is possible that relevant articles have been missed due to the search strategy and selection criteria that were applied. Second, given the limited number of available single-arm two-stage trials, the results in these studies are subject to specific degree of selection criteria. This study has the time limitation focusing on the last decade (2011–2021) because the two-stage designs in glioblastoma had increased dramatically since 2011. Lastly, this study focuses on right implementation for the study design and sample size calculation. Better understanding on the study drug information and targeted molecular information might help the understanding of why the two-stage trials had been terminated after the stage 1 and ineffective after successful completion of both stages.

Future directions: Brain tumor has specific design issues and obstacles like the blood brain barrier, heterogeneous nature in glioblastoma, and lack of accrual and longer study duration in clinical trials [64–67]. Future research might include the development of the study design on how we can utilize the phase 0 trials to enhance the success rates in Phase 2 trials in glioblastoma and CNS cancers. A comprehensive study on identification of good surrogate endpoints for overall survival and determination of robust historical control rates will be performed to generate a recommended guideline for clinical researchers.

## Conclusions

Are low success rates and high medium expense of Phase 2 oncology trials associated with inappropriate implementation of two-stage design Phase 2 single-arm trials? Among 29 trials reviewed systematically, 12 trails (41%) appropriately provided key input parameters and sample size results from two-stage design implementation. Among appropriately implemented 12 trials, discouragingly only 3 trials (10%) explained the reference information of historical control rates. Appropriate selection on primary endpoint, transparency of historical control and experimental rates, right implementation for two-stage design and sample size calculation, potential incorporation of adaptive designs, and utilization of Phase 0 paradigm [65, 67–70] might help overcoming the challenges on glioblastoma therapeutic trials in Phase 2 trials.

## Data Availability

Not applicable.
